# Nodal Marginal Zone Lymphoma with Prominent Expansion of PD-1+ T-Follicular Helper Cells: A Persistent Diagnostic Challenge with a Heterogeneous Mutational Architecture

**DOI:** 10.3390/ijms27010051

**Published:** 2025-12-20

**Authors:** Stefania Crisci, Annarosaria De Chiara, Maria Oro, Maria Rivieccio, Annalisa Altobelli, Sara Mele, Letizia Sirica, Daniela Donnarumma, Matteo Bonanni, Annarosa Cuccaro, Alberto Fresa, Rosaria De Filippi, Antonio Pinto

**Affiliations:** 1 Hematology-Oncology and Stem Cell Transplantation Unit, Istituto Nazionale Tumori-IRCCS-Fondazione ‘G. Pascale’, Via Mariano Semmola 49, 80131 Naples, Italy; s.crisci@istitutotumori.na.it (S.C.); maria.oro@istitutotumori.na.it (M.O.); maria.rivieccio@istitutotumori.na.it (M.R.); annalisa.altobelli@istitutotumori.na.it (A.A.); s.mele@istitutotumori.na.it (S.M.); letizia.sirica@istitutotumori.na.it (L.S.); d.donnarumma@istitutotumori.na.it (D.D.); matteo.bonanni@istitutotumori.na.it (M.B.); annarosa.cuccaro@istitutotumori.na.it (A.C.); alberto.fresa@istitutotumori.na.it (A.F.); 2 Pathology Unit, Istituto Nazionale Tumori-IRCCS-Fondazione ‘G. Pascale’, 80131 Naples, Italy; a.dechiara@istitutotumori.na.it; 3 Department of Pharmacy, Università degli Studi Federico II, 80131 Naples, Italy; rosaria.defilippi@istitutotumori.na.it

**Keywords:** nodal marginal zone lymphoma, PD-1, T-follicular helper cells, next-generation sequencing, clonality assessment, spatial genetic variations

## Abstract

Nodal marginal zone lymphoma (NMZL) is an indolent B-cell lymphoma that may pose diagnostic challenges due to the absence of distinct markers. In rare atypical cases, an overabundance of PD1+ T follicular helper (TFH) cells in tumor tissue may mimic peripheral T-cell lymphoma (PTCL) of TFH origin, further complicating the diagnosis. A 72-year-old woman with progressive lymphadenopathy had a cervical lymph node biopsy showing a disrupted architecture with monomorphic nodules of CD20+/MNDA+ B-cells and a prominent central population of proliferating CD4+/PD1+ T-cells, initially suggestive of a PTCL-TFH. The bone marrow contained aggregates of CD20+ B-cells intermixed with CD3+/CD4+/PD1+ T-cells. Next-generation sequencing (NGS) revealed clonal immunoglobulin heavy-chain rearrangements in the lymph node and bone marrow, with T-cell receptor genes displaying a polyclonal pattern. Targeted NGS showed no PTCL-related alterations but identified NMZL-associated mutations with different distributions across lymph node and bone marrow compartments. NOTCH2 mutations (c.6418C>T; p.Gln2140*) were found in both tissues, while the (c.69+2T>A; p.?) TNFRSF14 gene mutation was only detected in the lymph node. The KMT2D gene displayed a frameshift variant in the lymph node (c.4801_4802delinsT; p.Arg1601Leufs*3) and an in-frame deletion (c.11756_11758del; p.Gln3919del) in the bone marrow. Notably, NGS and digital droplet PCR confirmed a TP53 frameshift mutation (c.902del; p.Pro301Glnfs*44) with a fractional abundance of 0.31% in the lymph node and a (c.742C>T; p.Arg248Trp) mutation (0.309%) in the bone marrow. Results underscore the importance of NGS-based clonality to diagnose NMZL with prominent PD1+ T-cell hyperplasia, and prompt further investigation into tissue-specific mutational signatures in these unusual cases.

## 1. Introduction

Marginal zone lymphoma (MZL) comprises a group of indolent lymphomas accounting for approximately 10% of all B-cell non-Hodgkin lymphoma cases [1].

Based on clinical presentation, immunobiological characteristics and associated molecular lesions, three main subtypes of MZL have been identified: extranodal mucosa-associated lymphoid tissue (MALT) lymphoma (50–70% of cases), splenic marginal zone lymphoma (SMZL) (20%), and nodal marginal zone lymphoma (NMZL) (10–15%) [2,3,4,5,6].

More recently, the complex biology of MZL taxonomy has been further clarified through multilevel analysis of tumor cells, mutational profiling, and structural genetic abnormalities, along with an upgrade of previous provisional entities [2,3,4,5]. Splenic MZL has been categorized as a distinct clinicopathological entity, while the MZL family currently includes Extranodal EMZL/MALT, Primary cutaneous PC-MZL, Nodal MZL, and Paediatric MZL [2,3,4,5]. This view is overall shared by the 5th World Health Organization Classification of Haematolymphoid Tumours and the 2022 edition of the International Consensus Classification [3,4,5].

Patients with NMZL typically exhibit widespread nodal involvement, without evidence of extranodal or splenic disease, apart from bone marrow (BM) involvement [7,8]. Despite the disseminated disease at presentation, the clinical course is usually indolent, although histological transformation may occur in up to 15% of cases [1,7,8,9]. However, assessing the prognosis of these patients is challenging due to the debated effectiveness of the Follicular Lymphoma International Prognostic Index (FLIPI) [8,10]. The newer MZL International Prognostic Index may be a more effective option for evaluating patients with NMZL [11].

Molecular studies have revealed a complex range of common and subtype-specific genetic alterations in MZL B-cells, which may affect tumor growth and interactions with immune cells in the lymphoma microenvironment, leading to immune suppression and influencing treatment efficacy [2].

The diagnosis itself of NMZL can sometimes be complex due to its morphological overlap with other low-grade B-cell lymphomas, as well as the lack of distinct genetic or immunophenotypic markers [6,8]. However, recent findings by Guo et al. indicate that tumor cells in MZL consistently express CD180, a type 1 single-pass transmembrane protein belonging to the Toll-like receptor family. This expression differentiates MZL from other B-cell lymphoproliferative disorders and may significantly aid in the differential diagnosis of this lymphoma [12].

Distinguishing NMZL with a high component of reactive T-cell infiltrates from lymphomas originating from T-follicular helper (TFH) cells is particularly challenging due to their similar histopathologic features [13,14,15,16]. In 1999, Campo et al. first identified NMZL cases with increased reactive T-cells, while Bob et al. described the significant extrafollicular expansion of programmed cell death protein 1 (PD1)-positive non-malignant T-cells in a subset of cases [13,14,15,16]. Egan et al. further characterized this phenomenon in a comprehensive series of NMZL cases, identifying abnormal PD1 distribution patterns in 66.6% of cases and highlighting its importance as a potential diagnostic pitfall [17,18].

PD1 is a common marker of germinal center TFH cells, and its strong expression is a hallmark of peripheral T-cell lymphomas (PTCLs) originating from TFH cells, particularly angioimmunoblastic T-cell lymphoma [15,16]. This can create significant diagnostic challenges, as the notable presence of PD1+ TFH cells in certain cases of NMZL may potentially result in the misdiagnosis of nodal PTCL-TFH [18,19,20]. A proper diagnostic distinction is essential, as treatment and prognosis differ greatly between these conditions.

Hurwitz et al. recently highlighted the importance of mutational and clonality studies in cases of NMZL with a significant PD1+ T-cell hyperplasia [21]. They showed that comprehensive genomic profiling, including mutations in TNFRSF14, NOTCH2, KMT2D, and BRAF genes, along with the molecular evidence of B-cell clonality, can lead to a definitive diagnosis in these challenging cases [21]. In this context, next-generation sequencing (NGS) offers improved sensitivity and specificity for the detection of immunoglobulin (IG) and T-cell receptor (TCR) gene rearrangements, providing a more accurate characterization of the clonal landscape compared to traditional polymerase chain reaction (PCR) methods [19,22].

We encountered a rare case of NMZL that featured significant hyperplasia of PD1+ TFH cells, which initially led us to consider a diagnosis of PTCL. The management of this patient to reach the correct diagnosis emphasized the diagnostic value of NGS-based clonality assessments for such complex cases. Notably, application of a targeted NGS mutational profiling using gene panels for B-cell and T-cell lymphoma revealed a complex mutational landscape. The analysis also revealed unexpected spatial differences in the mutational patterns of lymphoma cells expanding in nodal and bone marrow tissues, which may influence biopsy site selection and disease progression monitoring. We discuss our findings in the context of previous research on the immunobiology and mutational profiling of this uncommon presentation of NMZL.

## 2. Results

### 2.1. Case Summary

In March 2023, a 72-year-old woman was assessed for painless lateral cervical lymphadenopathy lasting four months. Her medical history included only mild, asymptomatic tricuspid regurgitation. She did not report B-symptoms, and her ECOG Performance Status was 0. The laboratory workup showed borderline lymphocytosis with a lymphocyte count of 4.8 × 10^9^/L. The patient tested positive for hepatitis B virus antibodies (HBcAb and HBeAb) but negative for HBV-DNA and hepatitis C virus markers. The patient also exhibited bilateral ill-defined lesions with irregular margins in the subscapular areas. The swellings were firm and painless; however, the skin on the right side displayed patchy areas of cutaneous dyschromia, extending to the mid-thoracic back. Computed tomography (CT) showed enlarged lymph nodes in the right submandibular, lateral cervical, and jugulocarotid regions, along with smaller nodes in the supraclavicular, infraclavicular, and superior mediastinal areas. Bilateral lymphadenopathy was noted in the lumbar aorta, iliac, and inguinal regions. Abdominal ultrasounds showed mild enlargement of the spleen (13.0 cm) and liver (9.52 cm) without focal lesions. A PET-CT scan revealed hypermetabolic activity in several lymph node regions, including bilateral laterocervical and submandibular nodes (SUVmax 17.5), right axillary nodes (SUVmax 2.2), lumbar aortic nodes (SUVmax 4.6), and bilateral iliac and inguinal nodes (SUVmax 7.2). Additionally, there were hypermetabolic subcutaneous nodular lesions in the left frontal–parietal, right temporal, and upper nuchal regions (SUVmax 10.8). A subsequent dermatologic consultation raised the clinical suspicion of elastofibroma dorsi, interpreting the cutaneous dyschromia as related to chronic friction or local inflammation. An excisional biopsy of a right lateral cervical lymph node was initially suggestive of either a follicular B-cell lymphoma or a nodal T-follicular helper cell lymphoma, follicular type (nTFHL-f) [3,4]. However, following multimodal histopathologic and molecular assessments, including a bone marrow (BM) biopsy, the final diagnosis was of stage IVA non-bulky NMZL. The patient showed no organ dysfunction or significant laboratory abnormalities, including normal serum immunoglobulin levels, indicating that immediate treatment was unnecessary. At her last follow-up in June 2025, she remained asymptomatic with no signs of lymphoma progression.

### 2.2. Histopathological and Immunophenotypic Findings

**Figure 1:**
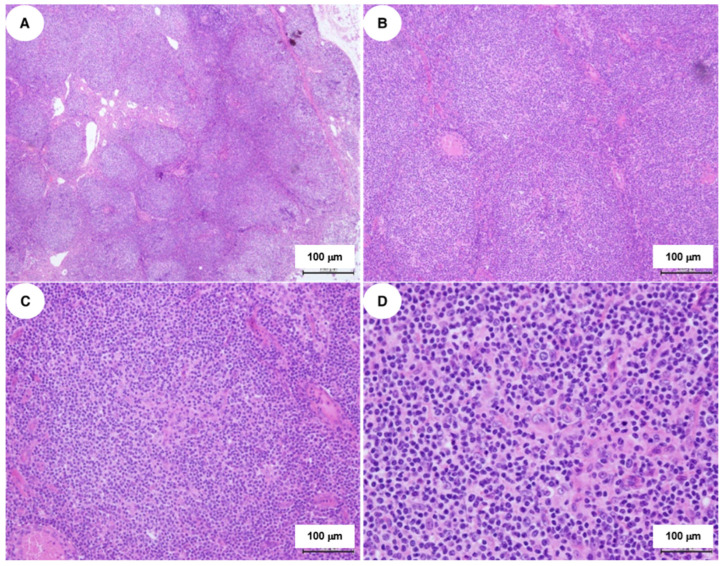
Hematoxylin and eosin-stained sections of the lymph node sample at varying magnifications. The images show the nodular architectural patterns and cellular morphology: (**A**) 4×; (**B**) 10×; (**C**) 20×; (**D**) 40×.

Histological examination of the lymph node showed disrupted architecture with multiple back-to-back monomorphic nodules (Figure 1).

**Figure 2:**
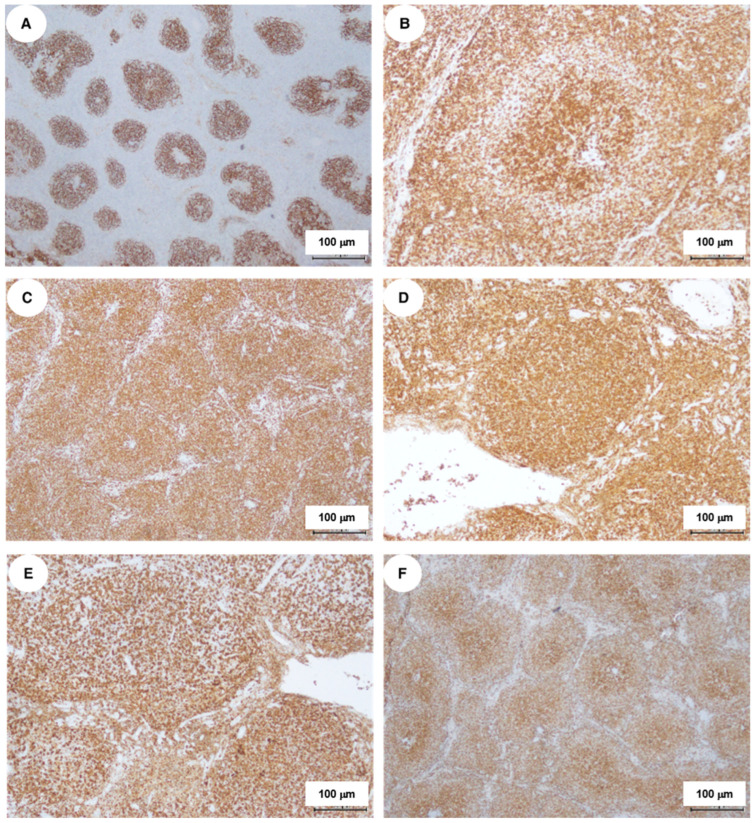
Results of immunostaining for the diagnostic lymph node sample: (**A**) CD21 staining demonstrates a well-preserved network of follicular dendritic cells. Immunolocalization and phenotype of the reactive T-cell population. (**B**) CD2; (**C**) CD3; (**D**) CD4; (**E**) CD5; (**F**) CD7.

Positive CD21 staining highlighted a well-preserved network of follicular dendritic cells (Figure 2A). The nodules were primarily composed of small lymphocytes with irregular nuclear contours and minimal cytoplasm. Scattered larger round cells exhibited immunoreactivity for CD2, CD3, CD5, and CD7, indicating a T-cell lineage (Figure 2B–F).

**Figure 3:**
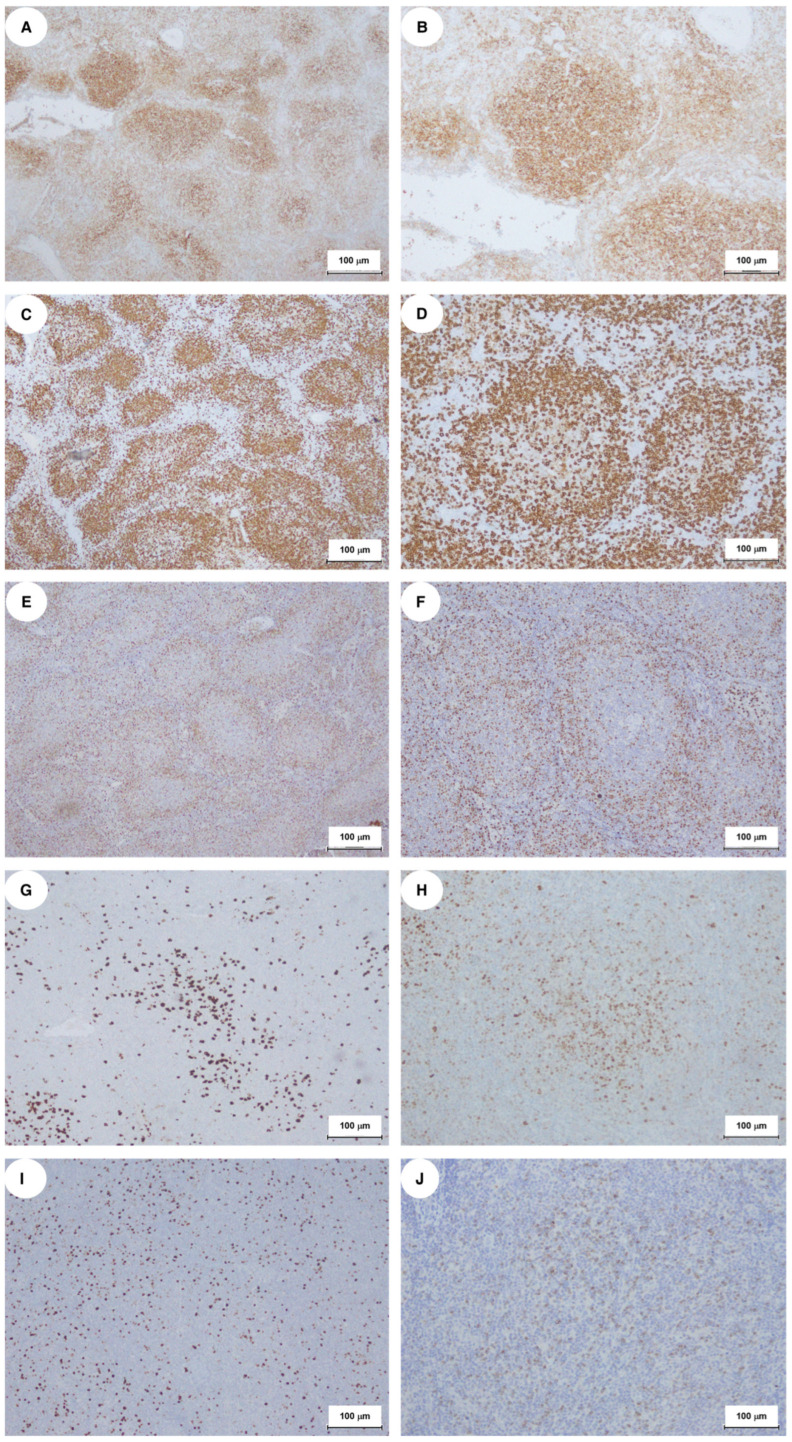
Immunohistochemical detection of key diagnostic markers indicating the B-cell phenotype of tumor cells, along with concurrent hyperplasia of reactive PD1+ T-lymphocytes exhibiting a THF phenotype: (**A**,**B**) PD1; (**C**,**D**) CD20; (**E**,**F**) MNDA; (**G**) CD10; (**H**) Bcl6; (**I**) Ki67; (**J**) TIA1.

The CD4/CD8 ratio was balanced, with more CD4+ T-cells in the center of nodules and CD8+ T-cells at the periphery. Central proliferating CD4+ T-cells expressed PD1 (Figure 3A,B) and occasionally CD30, but were negative for CD15, EBER, and HHV8. CD20+ and MNDA+ B-cells were primarily located at the follicle periphery (Figure 3C–F). Scattered cells tested positive for CD10 and BCL6, and the Ki67 proliferation index was 20%, indicating active proliferation in nodular regions (Figure 3G–I). Granzyme B was weak or absent, with variable positivity for TIA1 (Figure 3J).

**Figure 4:**
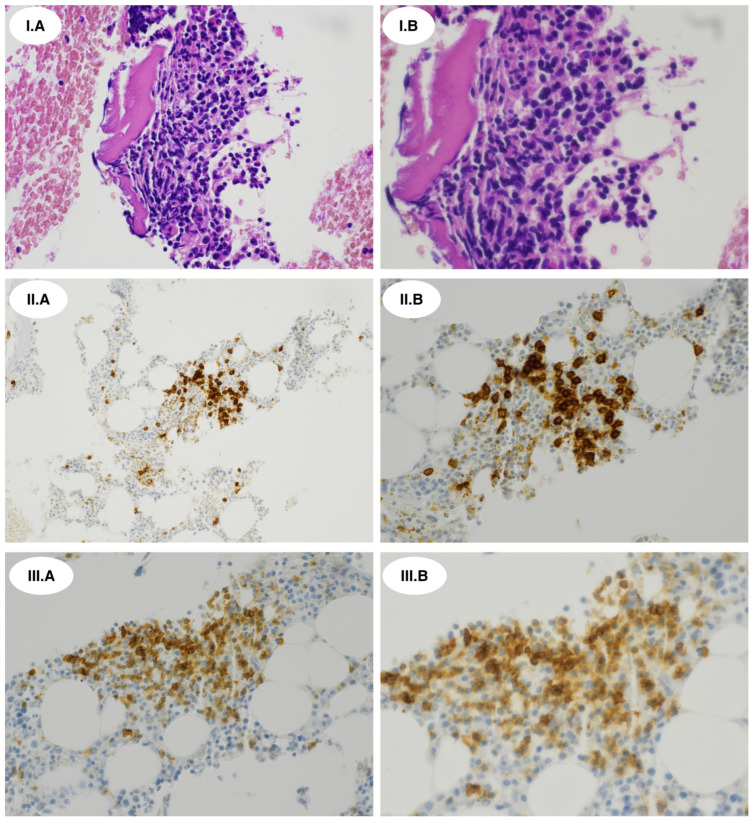
Morphological and immunohistochemical studies of bone marrow biopsy: (**I.A**,**I.B**) Hematoxylin and eosin staining shows infiltration with nodular lymphoid aggregates. (**II.A**,**II.B**) CD20 immunostaining highlights small lymphoma B-cells. (**III.A**,**III.B**) PD1 immunostaining reveals intermixed T-cells. For each staining, (**A**) original magnification 20×; (**B**) original magnification 40×.

The overall picture was suggestive of an NMZL with a “nodular central predominant pattern”. This pattern is observed in approximately 21% of NMZL cases and can complicate diagnosis due to its resemblance to nodular T-cell follicular lymphoma (nTFHL-f). Conventional PCR revealed clonal immunoglobulin heavy-chain (IGH) rearrangement, without common translocations such as t(14;18)(q32;q21)/IGH-BCL2 or t(11;18)(q21;q21)/IGH-BCL1. A BM biopsy revealed hypocellular areas (40% cellularity) with nodular aggregates of CD20+ small tumor B-cells intermixed with CD3+/PD1+ T-cells (Figure 4I.A–III.B).

Flow cytometry analysis of a BM aspirate showed a clonal CD19+ B-cell population with kappa light-chain restriction and a CD20+/CD22+/CD200+ CD79bdim profile, accounting for 25% of lymphocytes. T-cells, mainly of CD3+/CD4+ phenotype (60%), represented the majority of BM lymphocytes. PCR confirmed the clonal IGH rearrangement found in lymph node tissues, without detectable translocations.

### 2.3. Molecular Genetic Findings

#### 2.3.1. Clonality Assessment

**Figure 5:**
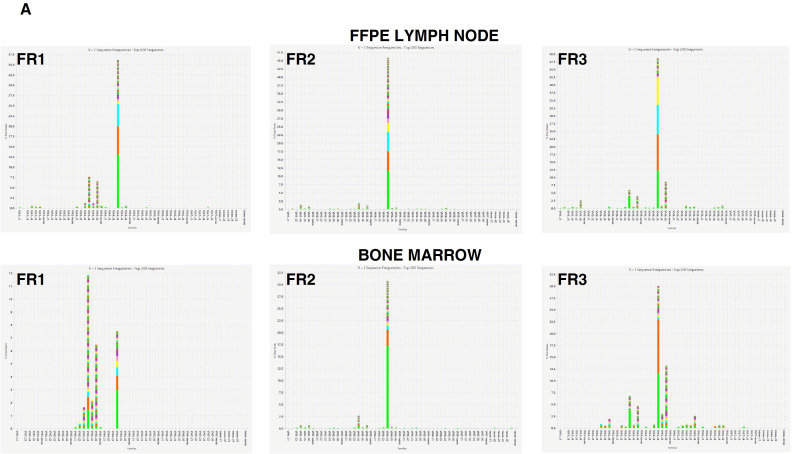

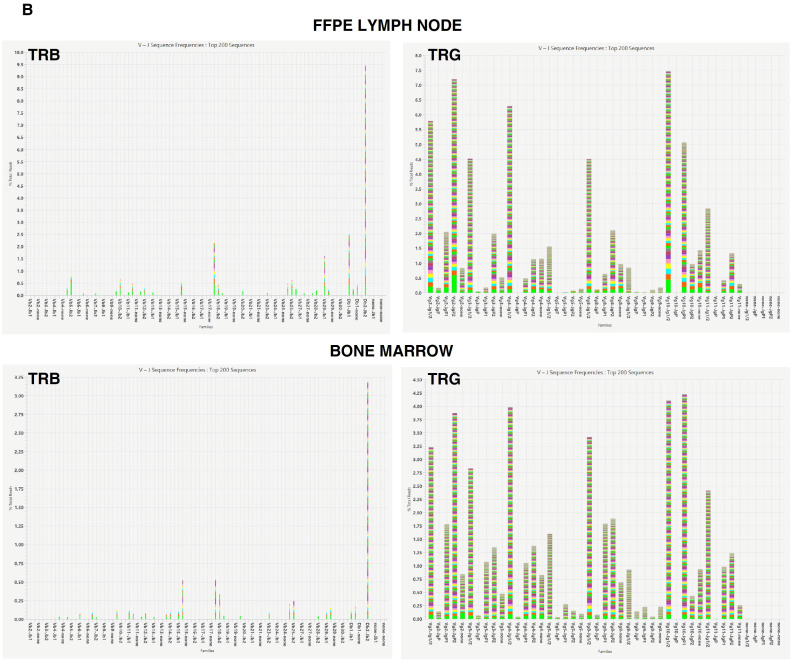
Next-generation sequencing-based assessment of B- and T-cell clonality in lymph node tissues and bone marrow. The images display clonal peaks for IGH rearrangement and a polyclonal pattern of TCR genes. Color-coded bars represent individual clonal sequences, with peak height indicating sequence frequency. (**A**) IGH rearrangement analysis (Framework1-FR1, Framework2-FR2, and Framework3-FR3) shows identical dominant clonal peaks in both samples. (**B**) TCR-beta and TCR-gamma analyses demonstrate polyclonal T-cell populations without evidence of clonality.

NGS analysis of FFPE lymph node tissue and BM cells confirmed B-cell clonality for IGH, with dominant rearrangements in both samples (Figure 5A). TCR analyses showed polyclonal T-cell populations (Figure 5B), ruling out a clonal T-cell process, including nTFHL-f, despite the observed PD1+ T-cell infiltrate.

#### 2.3.2. Mutational Profiling

Targeted NGS with B-cell and T-cell lymphoma gene panels was also diagnostically relevant, highlighting a complex mutational landscape in lymph node and BM samples. The T-cell lymphoma panel showed no mutations in RHOA, TET2, DNMT3A, IDH2, and other genes typically altered in TFH-derived and other T-cell lymphomas in both lymph node and BM specimens, further supporting the diagnosis of NMZL.

The B-cell panel revealed a complex, spatially diverse mutational landscape between lymph node and BM samples (Table 1).

**Figure 6:**
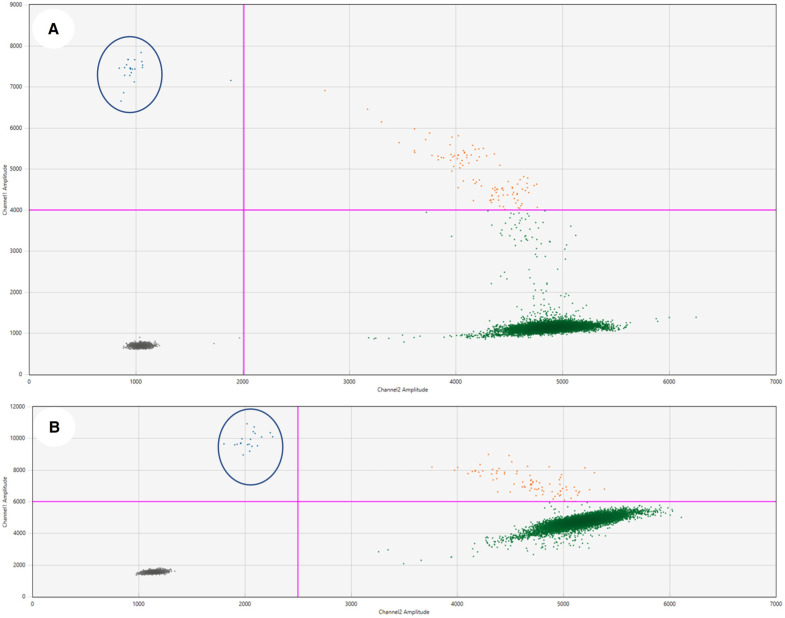
Digital droplet PCR analysis of TP53 mutations on FFPE lymph node tissue and bone marrow cells. Two-dimensional scatter plots generated by QuantaSoft™ Software 2.1.0.25 (Bio-Rad Laboratories, Inc., Hercules, CA, USA), display droplet fluorescence amplitudes across dual channels. Distinct color-coded clusters differentiate wild-type and mutant alleles, with mutation-positive droplets highlighted by blue circles. Threshold lines (pink line) for identifying positive droplets were established in accordance with the manufacturer’s specifications. Grey, green, blue, and orange dots represent background, HEX-positive, FAM-positive, and dual-positive signals, respectively: (**A**) Lymph node; TP53 c.902del frameshift mutation exhibiting a fractional abundance of 0.31%. (**B**) Bone marrow; TP53 c.742C>T missense mutation exhibiting a fractional abundance of 0.309%.

A nonsense mutation (c.6418C>T, p.Gln2140*) in the NOTCH2 gene was found in both lymph node tissue and BM cells, with variant allele frequencies (VAF) of 5.7% and 0.8%, respectively. Additionally, a splice site variant in the TNFRSF14 gene (c.69+2T>A; p?) was present only in the lymph node sample, with a VAF of 7.3%. This alteration, in terms of diagnostic relevance, likely disrupts the TNFRSF14 splice site, linked to NMZL and PD1+ T-cell hyperplasia. Two mutations in the KMT2D gene were identified: a frameshift variant in the lymph node (c.4801_4802delinsT; p.Arg1601Leufs*3) and an in-frame deletion (c.11756_11758del; p.Gln3919del) in BM cells. NGS analysis, validated by ddPCR, identified two distinct TP53 mutations with low VAFs in both lymph node and BM tissues. In the lymph node, we detected a TP53 c.902del frameshift mutation with a fractional abundance of 0.31% (Figure 6A). In the bone marrow, we observed a TP53 c.742C>T mutation with a fractional abundance of 0.309% (Figure 6B).

## 3. Discussion

Our report further emphasizes the need for a comprehensive integrated approach to differentiate between nodal NMZL with a significant expansion of reactive PD1+ T-cells and primary PTCL-TFH [13,14,15,16,17,18,19,21]. These uncommon cases pose a substantial diagnostic challenge because of the different treatment approaches and outcomes associated with each lymphoma entity [23,24]. PTCL-TFH typically requires anthracycline-based chemotherapy, possibly followed by high-dose therapy, while NMZL is often treated with less intensive regimens like single-agent rituximab or rituximab–bendamustine, with watchful waiting being an option in some cases [8,23,24]. Then, misdiagnosis can result in undertreatment or exposure to unnecessary toxicities. Despite the possible histopathologic mimicry and clinically pleomorphic presentation of PTCL, several clinical features help distinguish these T-cell tumors from NMZL. Due to their aggressive nature and distinct biology, patients with TFH-PTCL usually present with systemic B-symptoms, hyperinflammatory features, and autoimmune manifestations that are usually absent in NMZL. In addition, the presence of extranodal disease and of cutaneous involvement, including skin rashes or nodules, may suggest PTCL, while hepatomegaly and splenomegaly may occur in both entities. Therefore, some of the clinical features presented by our patient were unclear, which, along with the histopathologic findings, contributed to the initial diagnostic uncertainty [25,26,27].

**Figure 7:**
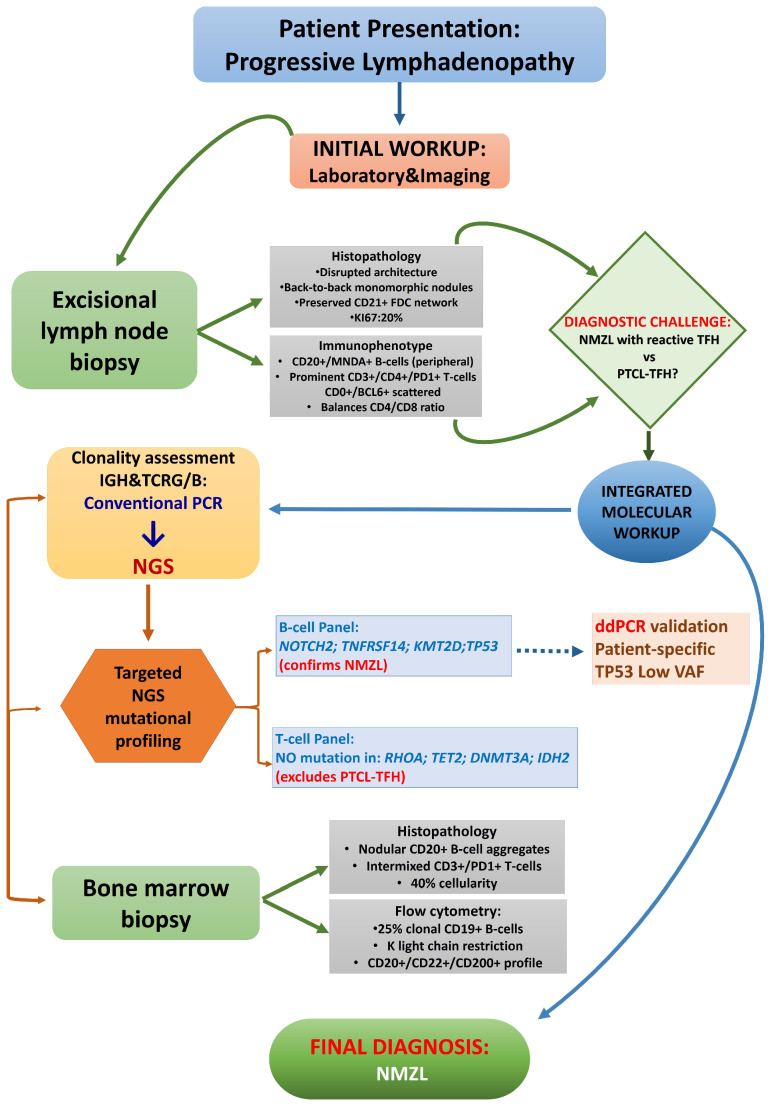
Diagnostic flowchart.

A dual approach addressed the diagnostic difficulties evidenced by prior studies [17,18,19,21]. NGS-based assessment effectively identified a clonal tumor B-cell population amid a significantly enlarged background of proliferating polyclonal T-cell populations. Likewise, disease-targeted NGS gene panels provided robust mutational evidence that confirmed B-cell origin and excluded T-cell malignancy. The NGS-based profiling not only facilitated an accurate diagnosis but also provided insights into the spatial genetic heterogeneity at various disease-related sites in these rare variants of NMZL. Our diagnostic flowchart is summarized in Figure 7.

We observed a complex mutational landscape with distinct differences between lymph nodes and BM. Key findings included shared mutations with varying VAFs in NOTCH2, site-specific alterations in TNFRSF14 in lymph nodes, and distinct TP53 and KMT2D mutations across disease-involved tissue compartments.

The nonsense mutation (c.6418C>T, p.Gln2140*) in the NOTCH2 gene is particularly relevant in this context. While mutations in the NOTCH2 gene are commonly found in MZL, our patient exhibited a loss-of-function mutation characterized as a “double-degron” mutation [2,28]. This mutation simultaneously disrupts two distinct degron motifs: the proline/glutamate/serine/threonine (PEST)-rich domain and the KLHL6-binding motif. As a result, this molecular alteration leads to increased stability and activity of the NOTCH2 protein compared to both the wild-type gene and single degron mutations [28]. Notably, the expression of the NOTCH2 Q2140* mutant in tumor cells of diffuse large B-cell lymphoma has been shown to promote resistance to CHOP chemotherapy in animal models [28]. In addition, the differential VAFs of NOTCH2 mutations in nodal and BM sites (5.7% vs. 0.8%) may reflect distinct microenvironmental pressures and/or subclonal evolution during disease dissemination [29,30].

The TNFRSF14 mutation (c.69+2T>A) found in the lymph node sample has previously been associated with the presence of a tumor-supportive microenvironment in follicular lymphoma [31,32]. This was explained by an increased local recruitment of TFH cells, likely due to impaired HVEM signaling [33]. Since PD1+ TFH cells are spatially associated with tumor B-cells in MZL tissues, it has also been suggested that they may directly promote tumor cell proliferation through contact-dependent and cytokine-dependent mechanisms [31,32,33,34,35,36,37].

However, the indolent clinical course observed in our patient and previously reported cases does not fully support the link between a hyperplastic PD1+ TFH cell compartment and the risk of early disease progression in NMZL [17,21].

The KMT2D gene is the most frequently altered gene in NMZL, harboring mutations in about 30% of the cases [2,38]. In our patient, this gene displayed two distinct loss-of-function mutations at a high allelic frequency in the lymph node and BM, further highlighting the genetic evolution of tumor B-cells during disease dissemination.

The clinical significance of the site-specific subclonal TP53 alterations, confirmed by ddPCR, which we identified at low allelic frequencies (<1%) as distinct variants in lymph nodes (c.902del frameshift) and BM (c.742C>T missense), remains to be established in MZL. Low-burden subclonal mutations in the TP53 gene have been identified in various B-cell malignancies, including chronic lymphocytic leukemia, mantle cell lymphoma, and follicular lymphoma [39,40]. While these mutations have been suggested as potential biomarkers for the early detection of more aggressive subclones that potentially drive disease evolution and progression, available data indicate that their prognostic significance should be assessed in conjunction with other clinicopathologic factors [2]. The presence of distinct TP53 subclonal mutations in lymph nodes and bone marrow from our patient highlights the importance of multi-site molecular assessments during follow-up and disease monitoring.

Overall, our molecular analyses are unlikely to be affected by variations in tumor B-cell content among the samples, since histopathology and flow cytometry documented that the clonal B-cell population in the BM significantly exceeded the sensitivity thresholds of the techniques utilized [41].

The genetic landscape of NMZL with prominent PD1+ THF cells expansion is not yet well established, mostly due to the rarity of these cases (Table 2). To our knowledge, about 27 cases of NMZL with THF cells hyperplasia were described, but mutational analysis has been performed on a limited fraction of the patients (Table 2) [17,18,19,21].

In these cases, mutations in NOTCH2, KMT2D, TNFAIP3, EZH2, KLF2, and TP53 genes, along with deletions involving CREBBP, KMT2D, and TNFRSF14, were detected [18,21]. While the mutational profile of our patient is generally consistent with previous findings, we describe the presence of a previously unreported NOTCH2 (p.Gln2140*) mutation and emphasize the still poorly explored site-specificity of certain molecular lesions.

NMZL has a higher mutational load than other forms of MZL, with recurrent mutations in MLL2, PTPRD, NOTCH2, KLF2, KMT2D, TNFAIP3, CREBBP, TNFRSF14, and BRAF genes [2,19,30,37,38,42,43]. While the prognostic significance of these alterations, including the risk of transformation into large B-cell lymphoma, requires further validation through prospective studies, their detection can assist in diagnosing ambiguous cases like the one presented here [19,37,38,42,43]. The clinical implementation of circulating cell-free DNA-based molecular genotyping is expected to provide additional relevant insights into the heterogeneous genetic landscape of these lymphomas [44].

The effective use of NGS-based clonality assessment and targeted mutation profiling in a challenging diagnostic case necessitates balancing cost-effectiveness with diagnostic accuracy [45,46]. While NGS has higher upfront costs than conventional PCR methods, its comprehensive nature may provide a critical value by preventing misdiagnosis that could lead to inappropriate therapeutic selection with substantial clinical and economic consequences [47]. NGS may prove particularly advantageous when applied to FFPE samples with suboptimal DNA quality due to formalin fixation and prolonged storage [48], enabling simultaneous IG/TCR clonality assessment and comprehensive mutational profiling in a single workflow, thereby reducing the need for multiple sequential diagnostic tests [43,46,49]. Unlike ddPCR, which requires prior knowledge of specific mutations, NGS provides an unbiased, broad-spectrum approach that identifies clinically relevant mutations (NOTCH2, TNFRSF14, KMT2D, TP53), informing diagnosis and prognosis [50]. Significantly, NGS facilitates identification of patient-specific molecular markers for subsequent cost-effective ddPCR-based longitudinal monitoring [51], enabling sensitive detection of residual lymphoproliferative disease and early detection of clonal evolution and relapse [51]. Advances in genomic technologies, including NGS and ddPCR, have now rendered extended genomic analyses of hematologic malignancies technologically and financially feasible for clinical application. As NGS costs continue to decrease with evolving reimbursement frameworks, this integrated diagnostic-to-monitoring strategy represents a rational approach balancing diagnostic precision with mid- and long-term efficiency [45].

## 4. Materials and Methods

### 4.1. Histopathology and Immunohistochemistry

Lymph node tissue was fixed in 10% neutral-buffered formalin, paraffin-embedded (FFPE) (Diapath processor, Martinengo, Italy), sectioned at 3 µm, stained with hematoxylin-eosin (Diapath), and coverslipped with Eukitt (Bio-Optica, Milano, Italy). For immunohistochemistry, sections were deparaffinized, rehydrated, and subjected to heat-induced epitope retrieval (pH 9.0 buffer, 30 min). Immunostaining was performed on a Ventana Benchmark Ultra autostainer (UltraView detection; Roche Diagnostics, Tucson, AZ, USA) with antibodies to CD20, CD2, CD3, CD4, CD5, CD7, CD10, CD21, BCL6, Ki-67, MNDA, PD-1, and additional markers (Supplementary Table S1). PD-1 expression was scored according to Egan et al. [17,52]. Immunohistochemistry for BM trephine biopsies was performed according to previously published procedures [53].

### 4.2. DNA Isolation and Quality Control

Genomic DNA was extracted from a fresh BM aspirate (Maxwell 16 LEV Blood DNA Kit) and 10 μm FFPE sections (Maxwell CSC DNA FFPE Kit) using a Maxwell CSC instrument (Promega, Milan, Italy). DNA yield was quantified by Qubit 4 fluorometry (Thermo Fisher Scientific, Waltham, MA, USA); integrity was assessed on an Agilent 4200 TapeStation (Santa Clara, CA, USA). DNA quality was validated using the Specimen Control Size Ladder assay (Invivoscribe, San Diego, CA, USA), based on EuroClonality/BIOMED-2 criteria [22].

### 4.3. Clonality Assessment

After conventional PCR assays, immunoglobulin heavy-chain (IGH, frameworks 1–3) and T-cell receptor γ/β (TRG/TRB) rearrangements were amplified with the LymphoTrack Assays Panels-MiSeq (Invivoscribe Technologies, Inc.) and sequenced on an Illumina MiSeqDx platform (Illumina, San Diego, CA, USA). Clonality interpretation was performed according to EuroClonality guidelines [22,54,55].

### 4.4. Targeted Next-Generation Sequencing

Mutational profiling employed two complementary panels: the 54-gene SOPHiA Lymphoma Solution (SOPHiA Genetics, Saint-Sulpice, Switzerland) targeting B-cell lymphomas, and a 53-gene custom panel (Illumina TruSight) for T-cell lymphomas (Supplementary Tables S2 and S3). Libraries were prepared from 50–300 ng DNA and sequenced on an Illumina MiSeqDx (2 × 150 bp). Sequencing data were analyzed using the SOPHiA Data Driven Medicine (DDM) platform (v5.10.54.3) and Alamut Visual Plus V1.13 (SOPHiA GENETICS), aligned to GRCh37/hg19. Variants were filtered for exonic non-synonymous changes, indels, or splice-site alterations with VAF ≥ 4.0% and classified according to ACMG criteria.

### 4.5. Droplet Digital PCR

Low-frequency TP53 mutations were validated using custom ddPCR assays on a QX200 Droplet Digital PCR (ddPCR) System (Bio-Rad Technologies, Hercules, CA, USA). Reactions contained 150 ng DNA, specific primers/probes (Supplementary Table S4), and ddPCR SuperMix for Probes (no dUTP). Mutant allele frequencies were calculated using QuantaSoft software v.2.1.0.25.

#### Limitations

Our study is based on a comprehensive analysis of a single patient due to the extreme rarity of such cases. Therefore, our conclusions should be interpreted with caution, even though they may serve as a foundation for generating hypotheses that could prompt further research into these uncommon NMZL variants. We also acknowledge that differences in tumor B-cell content among the examined samples, specifically between lymph node tissue and BM, may have potentially influenced our findings. However, we are confident that our molecular analyses are unlikely to be affected by this factor, as histopathological assessments and flow cytometry have demonstrated that the clonal B-cell population in the BM significantly exceeded the sensitivity thresholds of all the techniques employed [41].

## 5. Conclusions

Our report demonstrates that combining clonality assessment with targeted sequencing offers the most comprehensive diagnostic approach for challenging cases of NMZL with PD1+ TFH cell hyperplasia. Despite having a complex mutational profile, our patient experienced an indolent course, showing no signs of disease progression or transformation into aggressive lymphoma. This suggests that, in these cases, the endogenous immune control contrasting tumor progression is somehow preserved. Additionally, the heterogeneous mutational profile observed between tumor cells in the lymph nodes and those in the bone marrow emphasizes the importance of multi-site molecular assessments for follow-up and disease monitoring. Our report prompts further investigation into the genetic and microenvironmental factors that may drive disease evolution in these unusual variants of NMZL.

## Figures and Tables

**Table 1:** Comparative genetic analysis (lymph node vs. bone marrow) in a case of NMZL with hyperplasia of reactive PD1+TFH cells.

Lymph Node				Bone Marrow			
Gene Reference Sequence Exons	Chromosome Position	cDNA Protein Mutation Type	VAF (%)	Gene Reference Sequence Exons	Chromosome Position	cDNA Protein Mutation Type	VAF (%)
NOTCH2 NM_024408 34	1 120458927	c.6418C>T p.Gln2140* Nonsense	5.7	NOTCH2 NM_024408 34	1 120458927	c.6418C>T p.Gln2140* Nonsense	0.8
TNFRSF14 NM_001297605 1	1 2488174	c.69+2T>A p.? Splice Site	7.3	TNFRSF14 NM_001297605 1	NDA	NDA	NDA
KMT2D NM_003482 19	12 49438688	c.4801_4802delinsT p.Arg1601Leufs*3 Frameshift	5.5	KMT2D NM_003482 39	12 49426729	c.11756_11758del p.Gln3919del Deletion	5.7
TP53 NM_000546 8	17 7577035	c.902del p.Pro301Glnfs*44 Frameshift	0.3	TP53 NM_000546 7	17 7577539	c.742C>T p.Arg248Trp Missense	0.3

Chromosome coordinates are based on the Genome Reference Consortium Human Build 37 (GRCh37) genome assembly, with variant positions aligned to the hg19 reference genome. VAF, variant allele frequency; NDA, no detectable alteration.

**Table 2:** Details on immunopathology and mutational profiling of reported cases of NMZL with hyperplasia of reactive PD1+ T follicular helper cells.

Reference	Cases (*n*)	PD1 Staining Pattern	Molecular Methods	B/T-Cell Clonality	Key Mutations
Egan et al. 2020 [17]	14	Follicular Pattern (58%); Diffuse Pattern (8%); Reactive Pattern or Reduced Staining (33%)	sPCR-based clonality	IGH monoclonal TCR polyclonal	NA
Hurwitz et al. 2021 [21]	3	Follicular central (*n* = 1); Mixed follicular (central) and diffuse (*n* = 1); Mixed follicular (peripheral) and diffuse (*n* = 1)	sPCR-based clonality; Targeted NGS	IGH monoclonal TCR polyclonal	FC: NOTCH2 (FS), CREBBP (DEL), KLF2 (MS) MFD: NOTCH2 (NS), KLF2 (MS) MFPD: EZH2 (MS), TNFAIP3 (FS), TP53 (MS)
Deng et al. 2023 [18]	3	Reactive-like pattern (*n* = 1); Follicular pattern peripheral and central mixed (*n* = 1); Diffuse pattern tLBCL (*n* = 1)	sPCR-based clonality; Targeted NGS	IGH monoclonal TCR polyclonal	NOTCH2 (1/3; FS), KMT2D (3/3; DEL, FS, MS), TBL1XR1 (1/3; MS, SG), TNFAIP3 (2/3; FS), TNFRSF14 (1/3; DEL), EP300 (1/3; MS), KMT2C (1/3, MS), CD58 (1/3; MS)
Zamò et al. 2023 [19]	8	Nodular aggregates (75%) Diffuse pattern (25%)	Targeted NGS; clonality methods NA	IGH monoclonal TCR polyclonal	NOTCH2 (3/8), KLF2 (2/8), CREBBP (1/8), CD70 (1/8), IRF4 (1/8), SPEN (1/8), TMSB4X(1/8), BTG2(1/8), CCND3 (1/8), IRF8 (1/8), NOTCH1(1/8), TNFAIP3(1/8)
Present case	1	Predominantly central nodular	NGS-based clonality; Targeted NGS	IGH monoclonal TCR polyclonal	NOTCH2 (NS), TNFRSF14 (SS), KMT2D (FS, DEL), TP53 (FS, MS)

sPCR, standard polymerase chain reaction; IGH, immunoglobulin heavy chain gene rearrangement; TCR, T-cell receptor gene rearrangement; NA, not available; NGS, next-generation sequencing; FC, follicular central; MFD, mixed follicular (central) and diffuse; MFPD, mixed follicular (peripheral) and diffuse; FS, frameshift; DEL, deletion; MS, missense; NS, nonsense; SG, stop gain; SS, splice site; tLBCL, transformation into large B-cell lymphoma.

## Data Availability

The data presented in this study are openly available in [10.5281/zenodo.17672181] available at: https://doi.org/10.5281/zenodo.17672181.

## Supplementary Materials

The following supporting information can be downloaded at: https://www.mdpi.com/article/10.3390/ijms27010051/s1.
