# Supplementary material for: Nodal Marginal Zone Lymphoma with Prominent Expansion of PD-1+ T-Follicular Helper Cells: A Persistent Diagnostic Challenge with a Heterogeneous Mutational Architecture
Source: Int J Mol Sci. 2025 Dec 20;27(1):51. doi: 10.3390/ijms27010051 (PMC12785623; doi:10.3390/ijms27010051)
Supplement: Supplementary file 1 [file ijms-27-00051-s001.zip › ijms-4030348-supplementary.pdf]

Crisci *et al.*

Comprehensive Immunogenomic Characterization of Nodal Marginal Zone Lymphoma with Prominent PD-1+ T-Follicular Helper Cells Hyperplasia: Addressing a Persistent Diagnostic Challenge

SUPPLEMENTARY MATERIAL

Supplementary Methods

Histology and Immunohistochemistry

The excised lymph-node tissue was fixed in 10% neutral-buffered formalin and paraffin-embedded with a Diapath automatic processor. Sections (3 µm) were stained with hematoxylin–eosin (Diapath) following the manufacturer's protocol and mounted in Eukitt mounting medium (Bio-Optica) for microscopic evaluation.

For immunohistochemistry, paraffin was removed with xylene and sections were rehydrated through graded ethanols. Heat-induced epitope retrieval was performed in a pre-heated target-retrieval buffer (pH 9.0) for 30 min. Slides were processed on a Ventana Benchmark Ultra immunostainer with the UltraView detection system. The antibody panel comprised CD20, CD2, CD3, CD4, CD5, CD7, CD10, CD21, BCL6, Ki-67, MNDA, PD-1 and additional markers listed in Immunohistochemistry for BM threphine biopsies was performed according to previously published procedures [39].

Table S1. Staining patterns were evaluated and scored according to the criteria of Egan et al. for PD-1 distribution in nodal marginal-zone lymphoma [14, 38].

DNA Extraction and Quantification

Genomic DNA was extracted from bone-marrow aspirate with the Maxwell 16 LEV Blood DNA Kit and from 10 µm FFPE sections with the Maxwell CSC DNA FFPE Kit on a Maxwell CSC instrument (Promega Italia). DNA concentration was measured with the Qubit 1X dsDNA Broad-Range Assay on a Qubit 4 fluorometer. Fragment length and integrity were evaluated on an Agilent 4200

TapeStation with Genomic DNA ScreenTape. The Specimen Control Size Ladder assay (Invivoscribe), based on EuroClonality/BIOMED-2 Concerted Action BMH4-CT98-3936, was applied to confirm DNA suitability; a control master mix targeting Tbxas, RAG-1, PLZF and AF4 generated 100-, 200-, 300-, 400- and 600-bp amplicons.17 Only samples producing ≥ 400-bp products were advanced to sequencing.

Clonality Assessment by Next-Generation Sequencing

Clonal immunoglobulin and T-cell-receptor rearrangements were evaluated with LymphoTrack IGH (FR1/FR2/FR3), TRG and TRB Assay Panels (Invivoscribe Technologies, Inc). Each multiplex master mix (24 indexed reactions) was run with 50 ng input DNA. Amplicons were purified with AMPure XP beads (Beckman Coulter) and quantified on a QuantStudio 7 Pro real-time PCR system (Applied Biosystems). Sequencing employed an Illumina MiSeqDx with the appropriate reagent kit. The FASTq data output was analyzed using LymphoTrack MiSeq software, which determined DNA sequences, V-J assignments, sequence counts and frequencies of rearrangements. Clonality interpretation was performed following EuroClonality guidelines. For all panels the positive control has to be top percentage reads ≥2.5%, while the negative control top percentage reads <1.0%. Additionally, for MiSeq run validity, the %Q30 should exceed 75% for v2 (2 × 250 bp, only for LymphoTrack TRG Assay the %Q30 should exceed 80% for v2 (2 x 150 bp). The total number of reads for each sample is ≥20000. Clonal peaks were defined as those with a frequency >5% of total reads and at least 2.5-fold higher than the third most frequent peak per established guidelines [18, 40-41].

Targeted Next-Generation Sequencing

Mutational profiling was performed using two complementary NGS approaches. First, a targeted next-generation sequencing panel, SOPHiA Lymphoma Solution (SOPHiA GENETICS, Saint-Sulpice, Switzerland), was employed to analyze 54 genes associated with B-cell lymphomas, covering all coding regions of 32 genes and key hotspots of 22 genes (**Table S2**) for the detection of single-nucleotide variants, insertions/deletions, and gene amplifications. Library preparation utilized

50 ng genomic DNA with enzymatic fragmentation, end-repair, A-tailing, and adapter ligation according to manufacturer instructions. Following hybrid capture enrichment, libraries were amplified (12 PCR cycles) and purified using 0.8× AMPure XP beads. The custom T-cell lymphoma panel (Illumina DNA Prep with Enrichment-TruSight) covers 53 genes relevant to T-cell neoplasms (**Table S3**). Library preparation required 300 ng input DNA using the same workflow but with panel-specific capture probes. Both panels were sequenced on Illumina MiSeqDx using MiSeq Reagent Kit v3 (2×150 bp, 600 cycles). Multiplexed libraries were loaded at 12 pM with 1% PhiX control. Raw sequencing data from both panels were processed through the SOPHiA Data-Driven Medicine (v5.10.54.3) (DDM) proprietary bioinformatics pipeline and Alamut Visual Plus, aligned to GRCh37/hg19, and reviewed in Integrative Genomics Viewer (v2.6.3). Quality metrics demonstrated excellent coverage, with over 90% of mapped base pairs on-target, more than 99.99% of target regions achieving coverage greater than 500 reads, and 98.56% exceeding 1,000 reads. Variants were filtered for somatic exonic non-synonymous variants, small insertions/deletions, and splice site variants with a variant allele frequency (VAF) threshold of 4.0%. Variant classification followed the American College of Medical Genetics and Genomics (ACMG) criteria, incorporating prediction scores (SIFT, MutationTaster, PolyPhen-2), database annotations (dbSNP, ClinVar, OMIM, COSMIC, OncoKb, Franklin, VarSome), and population frequencies (gnomAD, G1000, ESP5400).

Droplet Digital PCR assay

Custom ddPCR assays were designed to confirm two low-frequency TP53 mutations (exon 8 c.902del, p.Pro301Glnfs*44 and exon 7 c.742C>T, p.Arg248Trp) identified by NGS. Primers and FAM/HEX-labeled probes were designed using Primer3Plus with optimized parameters for specific detection of mutant and wild-type alleles, yielding amplicons of 88 bp and 110 bp, respectively. Probe and primer details for all assays are presented in **Table S4**. Droplet digital PCR was performed using the QX200 AutoDG Droplet Digital PCR System (Bio-Rad Technologies, USA). Each 22 µL reaction contained 11 µL of 2× ddPCR SuperMix for Probes (no dUTP), template DNA, primers (900 nM

each), and probes (250 nM each). Following droplet generation, PCR amplification was performed (95°C for 10 min; 40 cycles of 94°C for 30 s and 55°C for 1 min; 98°C for 10 min). Droplet fluorescence was analyzed using the QX200 Droplet Reader, and QuantaSoft software (version 1.7.4) was used to calculate mutant allele frequencies by determining the ratio of FAM-positive (mutant) to HEX-positive (wild-type) droplets, with a detection limit of 0.1%.

SUPPLEMENTARY TABLES

Table S1. Antibodies used for Immunophenotyping. The table lists the specific antibodies utilized detailing the antigen, clone, dilution, and source for each antibody. The uniform use of PREDILUTE indicates consistent preparation across different staining protocols.

Antigen	Clone	Diluition	Source
**CD2**	MRQ-11	PREDILUTE	Ventana
**CD3**	2GV6	PREDILUTE	Ventana
**CD4**	SP35	PREDILUTE	Ventana
**CD5**	SP19	PREDILUTE	Ventana
**CD7**	SP94	PREDILUTE	Ventana
**CD8**	SP239	PREDILUTE	Ventana
**CD20**	L26	PREDILUTE	Ventana
**CD10**	SP67	PREDILUTE	Ventana
**Bcl6**	GI1917E/A8	PREDILUTE	Cell Marque
**Bcl2**	SP66	PREDILUTE	Ventana
**Ciclina D1**	SP4-R	PREDILUTE	Ventana
**CD21**	2G9	PREDILUTE	Ventana
**MNDA**	3C1	1:25	Cell Signaling
**Ki67**	30-9	PREDILUTE	Ventana
**PD1**	NAT105	PREDILUTE	Cell Marque
**TIA1**	2G9A1075	PREDILUTE	BIOGENEX
**CD30**	BER H2	PREDILUTE	Ventana
**CD15**	MMA	PREDILUTE	Ventana
**EBER-ISH**			Ventana
**HHV8**	13B10	PREDILUTE	Ventana

Table S2. Genes targeted by SOPHiA LYMPHOMA SOLUTION™ with analyzed exons indicated in brackets for each gene.

Lymphoma Solution® Panel
ARID1A, B2M, BCL2, CCND3, CD58, CHD2, CDKN2A, CDKN2B, CIITA, CXCR4, EP300, FOXO1, GNA13, ID3, IRF4, KMT2A, KMT2D, MAL, MEF2B, MYC, MYD88, NFKBIE, PAX5, PIM1, POT1, PRDM1, PTPN11, REL, SOCS1, TNFAIP3, TNFRSF14, TP53, ATM (57-63), BCL6 (8,9), BIRC3 (all,ex.2), BRAF (15), BTK (15), CARD11 (4-9), CCND1 (1), CD79A (4,5), CD79B (5,6), CREBBP (27-30), EZH2 (16,18), FBXW7 (9,10), KRAS (2,3), NOTCH1 (34), NOTCH2 (26-28,34), NRAS (2,3), PLCG2 (17-23), PTEN (5), SF3B1 (14,15), STAT6 (9-14), TCF3 (17-19), XPO1 (15-18)

Table S3. Genes targeted by the 53-gene custom panel (Illumina DNA Prep with Enrichment-TruSight). This panel was specifically designed to investigate mutations typically associated with T cell-derived lymphomas.

Illumina DNA Prep with Enrichment-TruSight cusom panel
ARID1A, ASXL3, ATM, BCOR, BCORL1, CARD11, CCR4, CD28, CDKN2A, CDKN2B, CDKN2C, CDKN2D, CREBBP, DDX3X, DNMT3A, DNMT3B, DUSP22, ECSIT, EP300, FOXO1, FYN, HDAC9, IDH2, JAK1, JAK2, JAK3, KDM6A, KIT, KMT2A, KMT2B, KMT2C, KMT2D, KRAS, NCOR2, NOTCH1, NOTCH3, NRAS, PDCD1, PLCG1, PRDM1, PRKCB, RHOA, SETD1B, SETD2, STAT3, STAT5A, STAT5B, TET2, TNFRSF1B, TP53, TP63, VAV1, YTHDF2

Table S4. Custom ddPCR assay design for TP53 mutation detection. The table details the primer and probe sequences used for the detection of two TP53 mutations (exon 8: c.902del, p.Pro301Glnfs*44 and exon 7: c.742C>T, p.Arg248Trp). For each assay, forward and reverse primers, wild-type (HEX-labeled) and mutant (FAM-labeled) probes, and amplicon sizes are reported.

Assay Characteristics	TP53 Exon 8 Assay	TP53 Exon 7 Assay
**Target Mutation**	c.902del, p.Pro301Glnfs*44	c.742C>T, p.Arg248Trp
**Forward Primer**	5'-GAGGAAGAGAATCTCCGCAAG-3'	5'-GTTGGCTCTGACTGTACCAC-3'
**Reverse Primer**	5'-CTTCTTGTCCTGCTTGCTTAC-3'	5'-CTGGAGTCTTCCAGTGTGAT-3'
**Wild-Type Probe (HEX)**	5'-CTGCCCCCAGGGAGCACTAA-3'	5'-CATGAACCGGAGGCCCATCCT-3'
**Mutant Probe (FAM)**	5'-CTGCCCCAGGGAGCACTAAG-3'	5'-CATGAACTGGAGGCCCATCCT-3'
**Amplicon Size**	88 bp	110 bp
